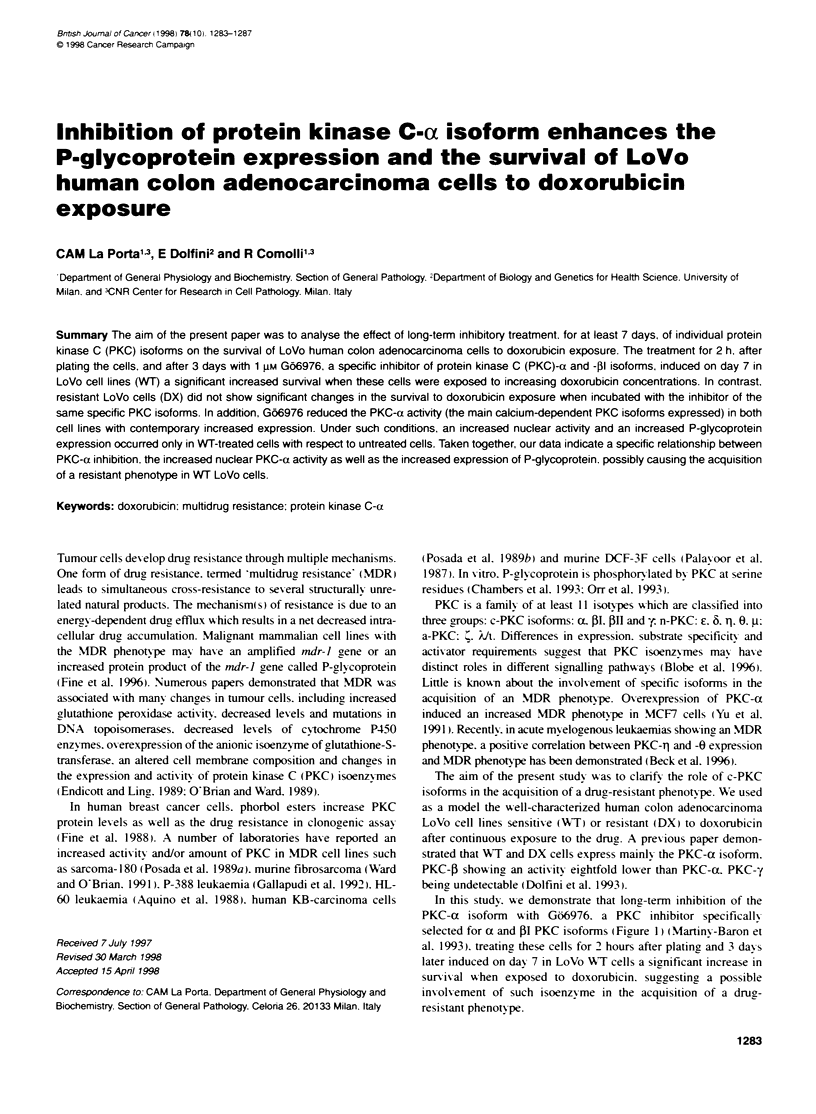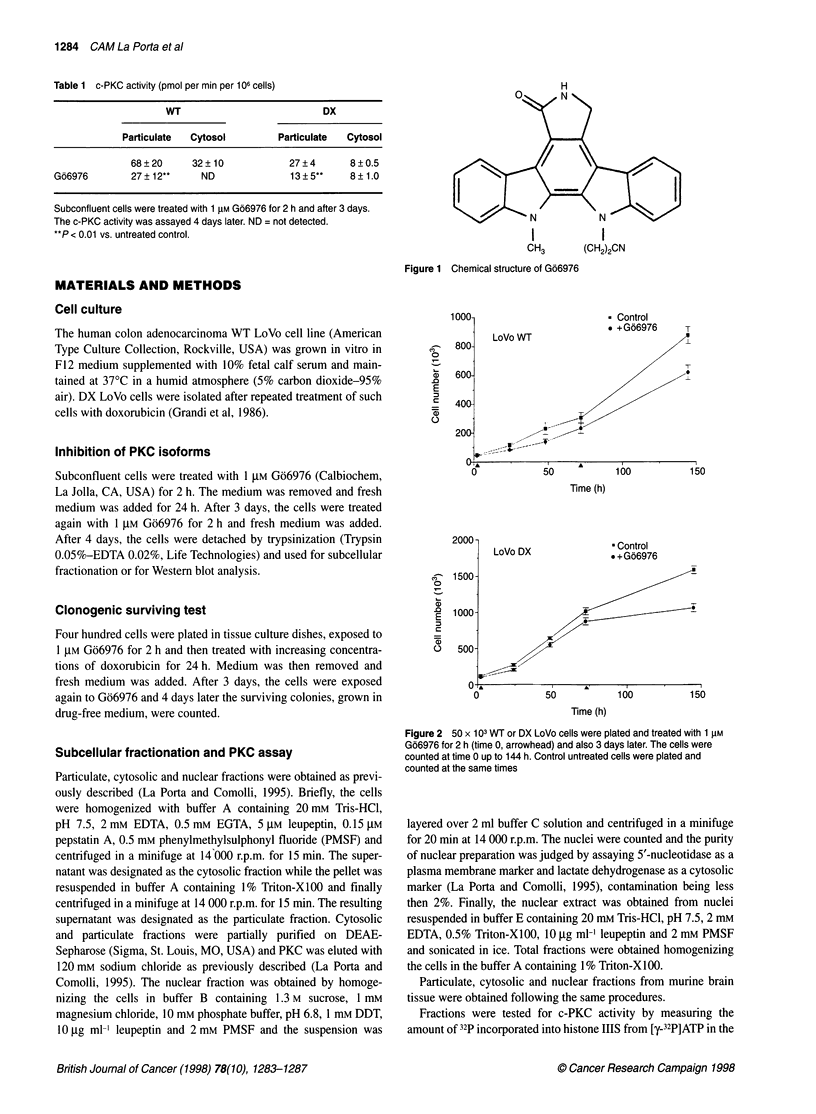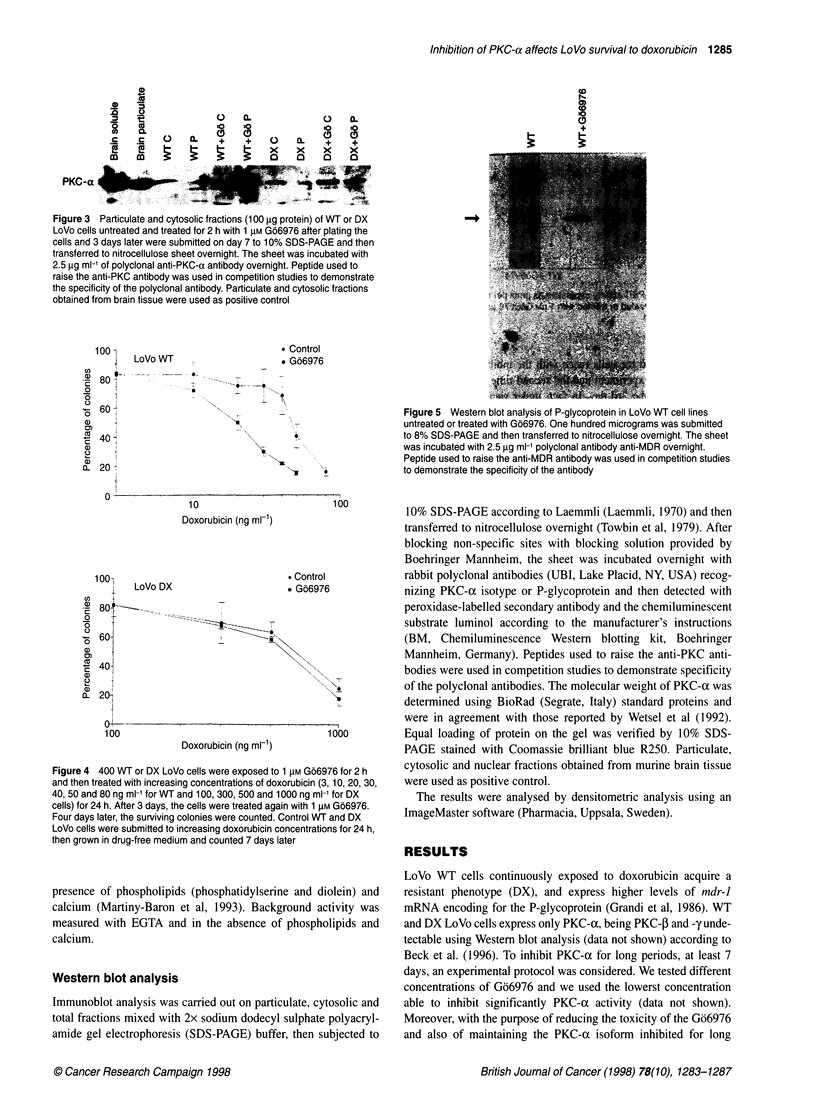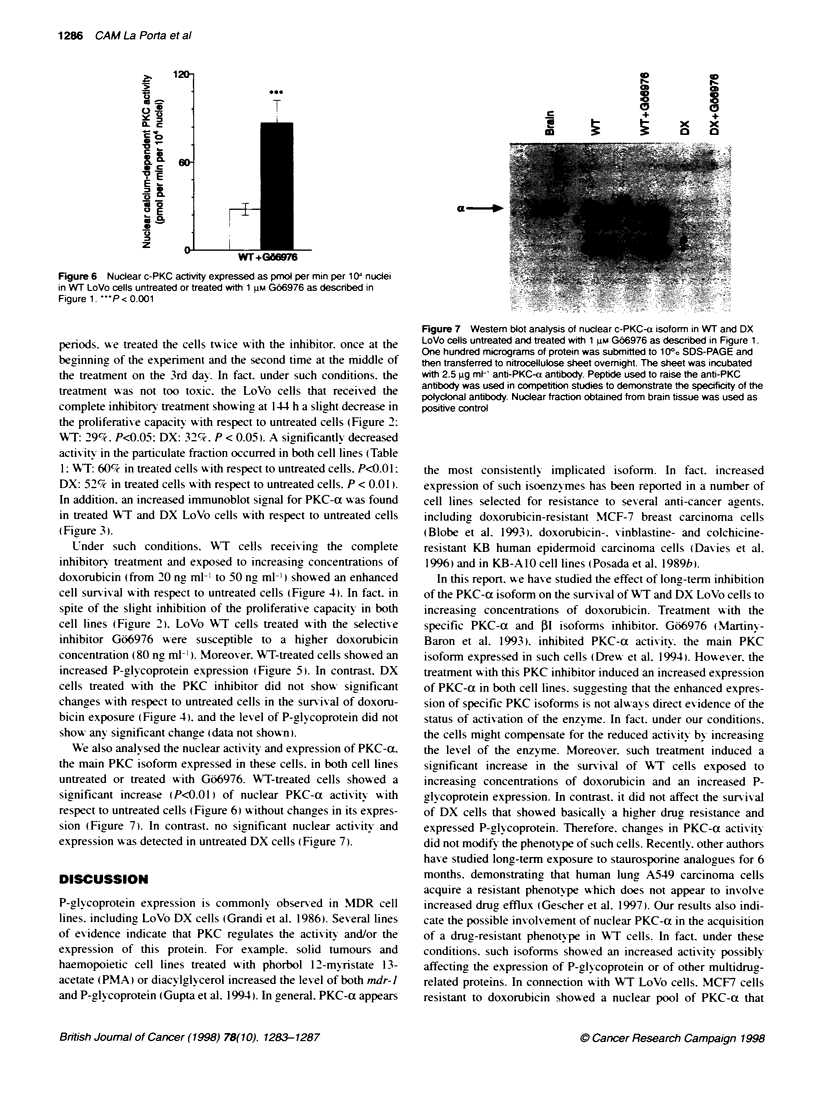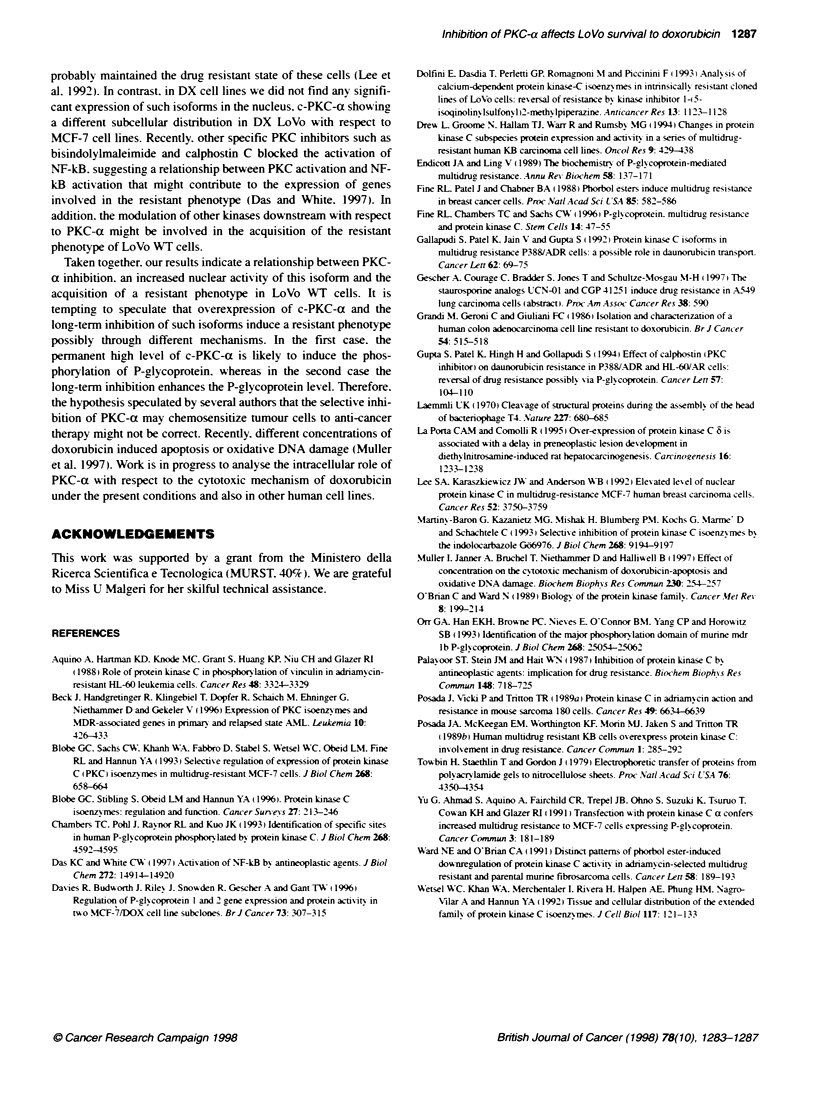# Inhibition of protein kinase C-alpha isoform enhances the P-glycoprotein expression and the survival of LoVo human colon adenocarcinoma cells to doxorubicin exposure.

**DOI:** 10.1038/bjc.1998.672

**Published:** 1998-11

**Authors:** C. A. La Porta, E. Dolfini, R. Comolli

**Affiliations:** Department of General Physiology and Biochemistry, University of Milan, Italy.

## Abstract

**Images:**


British Journal of Cancer (1998) 78(10), 1283–1287
© 1998 Cancer Research Campaign

### Inhibition of protein kinase C-α isoform enhances the P-glycoprotein expression and the survival of LoVo human colon adenocarcinoma cells to doxorubicin exposure

**CAM La Porta[1,3], E Dolfini[2] and R Comolli[1,3]**

[1]Department of General Physiology and Biochemistry, Section of General Pathology, [2]Department of Biology and Genetics for Health Science, University of Milan, and [3]CNR Center for Research in Cell Pathology, Milan, Italy

**Summary** The aim of the present paper was to analyse the effect of long-term inhibitory treatment, for at least 7 days, of individual protein kinase C (PKC) isoforms on the survival of LoVo human colon adenocarcinoma cells to doxorubicin exposure. The treatment for 2 h, after plating the cells, and after 3 days with 1 μM Gö6976, a specific inhibitor of protein kinase C (PKC)-α and -βI isoforms, induced on day 7 in LoVo cell lines (WT) a significant increased survival when these cells were exposed to increasing doxorubicin concentrations. In contrast, resistant LoVo cells (DX) did not show significant changes in the survival to doxorubicin exposure when incubated with the inhibitor of the same specific PKC isoforms. In addition, Gö6976 reduced the PKC-α activity (the main calcium-dependent PKC isoforms expressed) in both cell lines with contemporary increased expression. Under such conditions, an increased nuclear activity and an increased P-glycoprotein expression occurred only in WT-treated cells with respect to untreated cells. Taken together, our data indicate a specific relationship between PKC-α inhibition, the increased nuclear PKC-α activity as well as the increased expression of P-glycoprotein, possibly causing the acquisition of a resistant phenotype in WT LoVo cells.

**Keywords:** doxorubicin; multidrug resistance; protein kinase C-α

Tumour cells develop drug resistance through multiple mechanisms. One form of drug resistance, termed 'multidrug resistance' (MDR) leads to simultaneous cross-resistance to several structurally unrelated natural products. The mechanism(s) of resistance is due to an energy-dependent drug efflux which results in a net decreased intracellular drug accumulation. Malignant mammalian cell lines with the MDR phenotype may have an amplified *mdr-1* gene or an increased protein product of the *mdr-1* gene called P-glycoprotein (Fine et al, 1996). Numerous papers demonstrated that MDR was associated with many changes in tumour cells, including increased glutathione peroxidase activity, decreased levels and mutations in DNA topoisomerases, decreased levels of cytochrome P450 enzymes, overexpression of the anionic isoenzyme of glutathione-S-transferase, an altered cell membrane composition and changes in the expression and activity of protein kinase C (PKC) isoenzymes (Endicott and Ling, 1989; O'Brian and Ward, 1989).

In human breast cancer cells, phorbol esters increase PKC protein levels as well as the drug resistance in clonogenic assay (Fine et al, 1988). A number of laboratories have reported an increased activity and/or amount of PKC in MDR cell lines such as sarcoma-180 (Posada et al, 1989*a*), murine fibrosarcoma (Ward and O'Brian, 1991), P-388 leukaemia (Gallapudi et al, 1992), HL-60 leukaemia (Aquino et al, 1988), human KB-carcinoma cells (Posada et al, 1989*b*) and murine DCF-3F cells (Palayoor et al, 1987). In vitro, P-glycoprotein is phosphorylated by PKC at serine residues (Chambers et al, 1993; Orr et al, 1993).

PKC is a family of at least 11 isotypes which are classified into three groups: c-PKC isoforms: α, βI, βII and γ; n-PKC: ε, δ, η, θ, μ; a-PKC: ζ, λ/ι. Differences in expression, substrate specificity and activator requirements suggest that PKC isoenzymes may have distinct roles in different signalling pathways (Blobe et al, 1996). Little is known about the involvement of specific isoforms in the acquisition of an MDR phenotype. Overexpression of PKC-α induced an increased MDR phenotype in MCF7 cells (Yu et al, 1991). Recently, in acute myelogenous leukaemias showing an MDR phenotype, a positive correlation between PKC-η and -θ expression and MDR phenotype has been demonstrated (Beck et al, 1996).

The aim of the present study was to clarify the role of c-PKC isoforms in the acquisition of a drug-resistant phenotype. We used as a model the well-characterized human colon adenocarcinoma LoVo cell lines sensitive (WT) or resistant (DX) to doxorubicin after continuous exposure to the drug. A previous paper demonstrated that WT and DX cells express mainly the PKC-α isoform, PKC-β showing an activity eightfold lower than PKC-α, PKC-γ being undetectable (Dolfini et al, 1993).

In this study, we demonstrate that long-term inhibition of the PKC-α isoform with Gö6976, a PKC inhibitor specifically selected for α and βI PKC isoforms (Figure 1) (Martiny-Baron et al, 1993), treating these cells for 2 hours after plating and 3 days later induced on day 7 in LoVo WT cells a significant increase in survival when exposed to doxorubicin, suggesting a possible involvement of such isoenzyme in the acquisition of a drug-resistant phenotype.

*Received 7 July 1997*
*Revised 30 March 1998*
*Accepted 15 April 1998*

*Correspondence to:* CAM La Porta, Department of General Physiology and Biochemistry, Section of General Pathology, Celoria 26, 20133 Milan, Italy

Table 1 c-PKC activity (pmol per min per $10^6$ cells)

| | WT | | DX | |
|---|---|---|---|---|
| | Particulate | Cytosol | Particulate | Cytosol |
| | 68 ± 20 | 32 ± 10 | 27 ± 4 | 8 ± 0.5 |
| Gö6976 | 27 ± 12** | ND | 13 ± 5** | 8 ± 1.0 |

Subconfluent cells were treated with 1 μM Gö6976 for 2 h and after 3 days. The c-PKC activity was assayed 4 days later. ND = not detected.
**$P < 0.01$ vs. untreated control.

Figure 1 Chemical structure of Gö6976

### MATERIALS AND METHODS

### Cell culture

The human colon adenocarcinoma WT LoVo cell line (American Type Culture Collection, Rockville, USA) was grown in vitro in F12 medium supplemented with 10% fetal calf serum and maintained at 37°C in a humid atmosphere (5% carbon dioxide–95% air). DX LoVo cells were isolated after repeated treatment of such cells with doxorubicin (Grandi et al, 1986).

### Inhibition of PKC isoforms

Subconfluent cells were treated with 1 μM Gö6976 (Calbiochem, La Jolla, CA, USA) for 2 h. The medium was removed and fresh medium was added for 24 h. After 3 days, the cells were treated again with 1 μM Gö6976 for 2 h and fresh medium was added. After 4 days, the cells were detached by trypsinization (Trypsin 0.05%–EDTA 0.02%, Life Technologies) and used for subcellular fractionation or for Western blot analysis.

### Clonogenic surviving test

Four hundred cells were plated in tissue culture dishes, exposed to 1 μM Gö6976 for 2 h and then treated with increasing concentrations of doxorubicin for 24 h. Medium was then removed and fresh medium was added. After 3 days, the cells were exposed again to Gö6976 and 4 days later the surviving colonies, grown in drug-free medium, were counted.

### Subcellular fractionation and PKC assay

Particulate, cytosolic and nuclear fractions were obtained as previously described (La Porta and Comolli, 1995). Briefly, the cells were homogenized with buffer A containing 20 mM Tris-HCl, pH 7.5, 2 mM EDTA, 0.5 mM EGTA, 5 μM leupeptin, 0.15 μM pepstatin A, 0.5 mM phenylmethylsulphonyl fluoride (PMSF) and centrifuged in a minifuge at 14 000 r.p.m. for 15 min. The supernatant was designated as the cytosolic fraction while the pellet was resuspended in buffer A containing 1% Triton-X100 and finally centrifuged in a minifuge at 14 000 r.p.m. for 15 min. The resulting supernatant was designated as the particulate fraction. Cytosolic and particulate fractions were partially purified on DEAE-Sepharose (Sigma, St. Louis, MO, USA) and PKC was eluted with 120 mM sodium chloride as previously described (La Porta and Comolli, 1995). The nuclear fraction was obtained by homogenizing the cells in buffer B containing 1.3 M sucrose, 1 mM magnesium chloride, 10 mM phosphate buffer, pH 6.8, 1 mM DDT, 10 μg $ml^{-1}$ leupeptin and 2 mM PMSF and the suspension was

Figure 2 50 × $10^3$ WT or DX LoVo cells were plated and treated with 1 μM Gö6976 for 2 h (time 0, arrowhead) and also 3 days later. The cells were counted at time 0 up to 144 h. Control untreated cells were plated and counted at the same times layered over 2 ml buffer C solution and centrifuged in a minifuge for 20 min at 14 000 r.p.m. The nuclei were counted and the purity of nuclear preparation was judged by assaying 5′-nucleotidase as a plasma membrane marker and lactate dehydrogenase as a cytosolic marker (La Porta and Comolli, 1995), contamination being less then 2%. Finally, the nuclear extract was obtained from nuclei resuspended in buffer E containing 20 mM Tris-HCl, pH 7.5, 2 mM EDTA, 0.5% Triton-X100, 10 μg $ml^{-1}$ leupeptin and 2 mM PMSF and sonicated in ice. Total fractions were obtained homogenizing the cells in the buffer A containing 1% Triton-X100.

Particulate, cytosolic and nuclear fractions from murine brain tissue were obtained following the same procedures.

Fractions were tested for c-PKC activity by measuring the amount of $^{32}P$ incorporated into histone IIIS from [$\gamma$-$^{32}P$]ATP in the

© Cancer Research Campaign 1998

**Figure 3** Particulate and cytosolic fractions (100 μg protein) of WT or DX LoVo cells untreated and treated for 2 h with 1 μM Gö6976 after plating the cells and 3 days later were submitted on day 7 to 10% SDS-PAGE and then transferred to nitrocellulose sheet overnight. The sheet was incubated with 2.5 μg ml$^{-1}$ of polyclonal anti-PKC-α antibody overnight. Peptide used to raise the anti-PKC antibody was used in competition studies to demonstrate the specificity of the polyclonal antibody. Particulate and cytosolic fractions obtained from brain tissue were used as positive control

**Figure 4** 400 WT or DX LoVo cells were exposed to 1 μM Gö6976 for 2 h and then treated with increasing concentrations of doxorubicin (3, 10, 20, 30, 40, 50 and 80 ng ml$^{-1}$ for WT and 100, 300, 500 and 1000 ng ml$^{-1}$ for DX cells) for 24 h. After 3 days, the cells were treated again with 1 μM Gö6976. Four days later, the surviving colonies were counted. Control WT and DX LoVo cells were submitted to increasing doxorubicin concentrations for 24 h, then grown in drug-free medium and counted 7 days later presence of phospholipids (phosphatidylserine and diolein) and calcium (Martiny-Baron et al, 1993). Background activity was measured with EGTA and in the absence of phospholipids and calcium.

### Western blot analysis

Immunoblot analysis was carried out on particulate, cytosolic and total fractions mixed with 2× sodium dodecyl sulphate polyacrylamide gel electrophoresis (SDS-PAGE) buffer, then subjected to

**Figure 5** Western blot analysis of P-glycoprotein in LoVo WT cell lines untreated or treated with Gö6976. One hundred micrograms was submitted to 8% SDS-PAGE and then transferred to nitrocellulose overnight. The sheet was incubated with 2.5 μg ml$^{-1}$ polyclonal antibody anti-MDR overnight. Peptide used to raise the anti-MDR antibody was used in competition studies to demonstrate the specificity of the antibody

10% SDS-PAGE according to Laemmli (Laemmli, 1970) and then transferred to nitrocellulose overnight (Towbin et al, 1979). After blocking non-specific sites with blocking solution provided by Boehringer Mannheim, the sheet was incubated overnight with rabbit polyclonal antibodies (UBI, Lake Placid, NY, USA) recognizing PKC-α isotype or P-glycoprotein and then detected with peroxidase-labelled secondary antibody and the chemiluminescent substrate luminol according to the manufacturer's instructions (BM, Chemiluminescence Western blotting kit, Boehringer Mannheim, Germany). Peptides used to raise the anti-PKC antibodies were used in competition studies to demonstrate specificity of the polyclonal antibodies. The molecular weight of PKC-α was determined using BioRad (Segrate, Italy) standard proteins and were in agreement with those reported by Wetsel et al (1992). Equal loading of protein on the gel was verified by 10% SDS-PAGE stained with Coomassie brilliant blue R250. Particulate, cytosolic and nuclear fractions obtained from murine brain tissue were used as positive control.

The results were analysed by densitometric analysis using an ImageMaster software (Pharmacia, Uppsala, Sweden).

### RESULTS

LoVo WT cells continuously exposed to doxorubicin acquire a resistant phenotype (DX), and express higher levels of *mdr-1* mRNA encoding for the P-glycoprotein (Grandi et al, 1986). WT and DX LoVo cells express only PKC-α, being PKC-β and -γ undetectable using Western blot analysis (data not shown) according to Beck et al. (1996). To inhibit PKC-α for long periods, at least 7 days, an experimental protocol was considered. We tested different concentrations of Gö6976 and we used the lowerst concentration able to inhibit significantly PKC-α activity (data not shown). Moreover, with the purpose of reducing the toxicity of the Gö6976 and also of maintaining the PKC-α isoform inhibited for long

© Cancer Research Campaign 1998

**Figure 6** Nuclear c-PKC activity expressed as pmol per min per $10^4$ nuclei in WT LoVo cells untreated or treated with 1 μM Gö6976 as described in Figure 1. $^{***}P < 0.001$

**Figure 7** Western blot analysis of nuclear c-PKC-α isoform in WT and DX LoVo cells untreated and treated with 1 μM Gö6976 as described in Figure 1. One hundred micrograms of protein was submitted to 10% SDS-PAGE and then transferred to nitrocellulose sheet overnight. The sheet was incubated with 2.5 μg $ml^{-1}$ anti-PKC-α antibody. Peptide used to raise the anti-PKC antibody was used in competition studies to demonstrate the specificity of the polyclonal antibody. Nuclear fraction obtained from brain tissue was used as positive control periods, we treated the cells twice with the inhibitor, once at the beginning of the experiment and the second time at the middle of the treatment on the 3rd day. In fact, under such conditions, the treatment was not too toxic, the LoVo cells that received the complete inhibitory treatment showing at 144 h a slight decrease in the proliferative capacity with respect to untreated cells (Figure 2; WT: 29%, $P<0.05$; DX: 32%, $P < 0.05$). A significantly decreased activity in the particulate fraction occurred in both cell lines (Table 1; WT: 60% in treated cells with respect to untreated cells, $P<0.01$; DX: 52% in treated cells with respect to untreated cells, $P < 0.01$). In addition, an increased immunoblot signal for PKC-α was found in treated WT and DX LoVo cells with respect to untreated cells (Figure 3).

Under such conditions, WT cells receiving the complete inhibitory treatment and exposed to increasing concentrations of doxorubicin (from 20 ng $ml^{-1}$ to 50 ng $ml^{-1}$) showed an enhanced cell survival with respect to untreated cells (Figure 4). In fact, in spite of the slight inhibition of the proliferative capacity in both cell lines (Figure 2), LoVo WT cells treated with the selective inhibitor Gö6976 were susceptible to a higher doxorubicin concentration (80 ng $ml^{-1}$). Moreover, WT-treated cells showed an increased P-glycoprotein expression (Figure 5). In contrast, DX cells treated with the PKC inhibitor did not show significant changes with respect to untreated cells in the survival of doxorubicin exposure (Figure 4), and the level of P-glycoprotein did not show any significant change (data not shown).

We also analysed the nuclear activity and expression of PKC-α, the main PKC isoform expressed in these cells, in both cell lines untreated or treated with Gö6976. WT-treated cells showed a significant increase ($P<0.01$) of nuclear PKC-α activity with respect to untreated cells (Figure 6) without changes in its expression (Figure 7). In contrast, no significant nuclear activity and expression was detected in untreated DX cells (Figure 7).

### DISCUSSION

P-glycoprotein expression is commonly observed in MDR cell lines, including LoVo DX cells (Grandi et al, 1986). Several lines of evidence indicate that PKC regulates the activity and/or the expression of this protein. For example, solid tumours and haemopoietic cell lines treated with phorbol 12-myristate 13-acetate (PMA) or diacylglycerol increased the level of both *mdr-1* and P-glycoprotein (Gupta et al, 1994). In general, PKC-α appears the most consistently implicated isoform. In fact, increased expression of such isoenzymes has been reported in a number of cell lines selected for resistance to several anti-cancer agents, including doxorubicin-resistant MCF-7 breast carcinoma cells (Blobe et al, 1993), doxorubicin-, vinblastine- and colchicine-resistant KB human epidermoid carcinoma cells (Davies et al, 1996) and in KB-A10 cell lines (Posada et al, 1989*b*).

In this report, we have studied the effect of long-term inhibition of the PKC-α isoform on the survival of WT and DX LoVo cells to increasing concentrations of doxorubicin. Treatment with the specific PKC-α and βI isoforms inhibitor, Gö6976 (Martiny-Baron et al, 1993), inhibited PKC-α activity, the main PKC isoform expressed in such cells (Drew et al, 1994). However, the treatment with this PKC inhibitor induced an increased expression of PKC-α in both cell lines, suggesting that the enhanced expression of specific PKC isoforms is not always direct evidence of the status of activation of the enzyme. In fact, under our conditions, the cells might compensate for the reduced activity by increasing the level of the enzyme. Moreover, such treatment induced a significant increase in the survival of WT cells exposed to increasing concentrations of doxorubicin and an increased P-glycoprotein expression. In contrast, it did not affect the survival of DX cells that showed basically a higher drug resistance and expressed P-glycoprotein. Therefore, changes in PKC-α activity did not modify the phenotype of such cells. Recently, other authors have studied long-term exposure to staurosporine analogues for 6 months, demonstrating that human lung A549 carcinoma cells acquire a resistant phenotype which does not appear to involve increased drug efflux (Gescher et al, 1997). Our results also indicate the possible involvement of nuclear PKC-α in the acquisition of a drug-resistant phenotype in WT cells. In fact, under these conditions, such isoforms showed an increased activity possibly affecting the expression of P-glycoprotein or of other multidrug-related proteins. In connection with WT LoVo cells, MCF7 cells resistant to doxorubicin showed a nuclear pool of PKC-α that

© Cancer Research Campaign 1998

probably maintained the drug resistant state of these cells (Lee et al. 1992). In contrast, in DX cell lines we did not find any significant expression of such isoforms in the nucleus, c-PKC-α showing a different subcellular distribution in DX LoVo with respect to MCF-7 cell lines. Recently, other specific PKC inhibitors such as bisindolylmaleimide and calphostin C blocked the activation of NF-kB, suggesting a relationship between PKC activation and NF-kB activation that might contribute to the expression of genes involved in the resistant phenotype (Das and White, 1997). In addition, the modulation of other kinases downstream with respect to PKC-α might be involved in the acquisition of the resistant phenotype of LoVo WT cells.

Taken together, our results indicate a relationship between PKC-α inhibition, an increased nuclear activity of this isoform and the acquisition of a resistant phenotype in LoVo WT cells. It is tempting to speculate that overexpression of c-PKC-α and the long-term inhibition of such isoforms induce a resistant phenotype possibly through different mechanisms. In the first case, the permanent high level of c-PKC-α is likely to induce the phosphorylation of P-glycoprotein, whereas in the second case the long-term inhibition enhances the P-glycoprotein level. Therefore, the hypothesis speculated by several authors that the selective inhibition of PKC-α may chemosensitize tumour cells to anti-cancer therapy might not be correct. Recently, different concentrations of doxorubicin induced apoptosis or oxidative DNA damage (Muller et al. 1997). Work is in progress to analyse the intracellular role of PKC-α with respect to the cytotoxic mechanism of doxorubicin under the present conditions and also in other human cell lines.

### ACKNOWLEDGEMENTS

This work was supported by a grant from the Ministero della Ricerca Scientifica e Tecnologica (MURST, 40%). We are grateful to Miss U Malgeri for her skilful technical assistance.

### REFERENCES

Aquino A, Hartman KD, Knode MC, Grant S, Huang KP, Niu CH and Glazer RI (1988) Role of protein kinase C in phosphorylation of vinculin in adriamycin-resistant HL-60 leukemia cells. *Cancer Res* **48**: 3324–3329

Beck J, Handgretinger R, Klingebiel T, Dopfer R, Schaich M, Ehninger G, Niethammer D and Gekeler V (1996) Expression of PKC isoenzymes and MDR-associated genes in primary and relapsed state AML. *Leukemia* **10**: 426–433

Blobe GC, Sachs CW, Khanh WA, Fabbro D, Stabel S, Wetsel WC, Obeid LM, Fine RL and Hannun YA (1993) Selective regulation of expression of protein kinase C (PKC) isoenzymes in multidrug-resistant MCF-7 cells. *J Biol Chem* **268**: 658–664

Blobe GC, Stibling S, Obeid LM and Hannun YA (1996). Protein kinase C isoenzymes: regulation and function. *Cancer Surveys* **27**: 213–246

Chambers TC, Pohl J, Raynor RL and Kuo JK (1993) Identification of specific sites in human P-glycoprotein phosphorylated by protein kinase C. *J Biol Chem* **268**: 4592–4595

Das KC and White CW (1997) Activation of NF-kB by antineoplastic agents. *J Biol Chem* **272**: 14914–14920

Davies R, Budworth J, Riley J, Snowden R, Gescher A and Gant TW (1996) Regulation of P-glycoprotein 1 and 2 gene expression and protein activity in two MCF-7/DOX cell line subclones. *Br J Cancer* **73**: 307–315

Dolfini E, Dasdia T, Perletti GP, Romagnoni M and Piccinini F (1993) Analysis of calcium-dependent protein kinase-C isoenzymes in intrinsically resistant cloned lines of LoVo cells: reversal of resistance by kinase inhibitor 1-(5-isoqinolinylsulfonyl)2-methylpiperazine. *Anticancer Res* **13**: 1123–1128

Drew L, Groome N, Hallam TJ, Warr R and Rumsby MG (1994) Changes in protein kinase C subspecies protein expression and activity in a series of multidrug-resistant human KB carcinoma cell lines. *Oncol Res* **9**: 429–438

Endicott JA and Ling V (1989) The biochemistry of P-glycoprotein-mediated multidrug resistance. *Annu Rev Biochem* **58**: 137–171

Fine RL, Patel J and Chabner BA (1988) Phorbol esters induce multidrug resistance in breast cancer cells. *Proc Natl Acad Sci USA* **85**: 582–586

Fine RL, Chambers TC and Sachs CW (1996) P-glycoprotein, multidrug resistance and protein kinase C. *Stem Cells* **14**: 47–55

Gallapudi S, Patel K, Jain V and Gupta S (1992) Protein kinase C isoforms in multidrug resistance P388/ADR cells: a possible role in daunorubicin transport. *Cancer Lett* **62**: 69–75

Gescher A, Courage C, Bradder S, Jones T and Schultze-Mosgau M-H (1997) The staurosporine analogs UCN-01 and CGP 41251 induce drug resistance in A549 lung carcinoma cells (abstract). *Proc Am Assoc Cancer Res* **38**: 590

Grandi M, Geroni C and Giuliani FC (1986) Isolation and characterization of a human colon adenocarcinoma cell line resistant to doxorubicin. *Br J Cancer* **54**: 515–518

Gupta S, Patel K, Hingh H and Gollapudi S (1994) Effect of calphostin (PKC inhibitor) on daunorubicin resistance in P388/ADR and HL-60/AR cells: reversal of drug resistance possibly via P-glycoprotein. *Cancer Lett* **57**: 104–110

Laemmli UK (1970) Cleavage of structural proteins during the assembly of the head of bacteriophage T4. *Nature* **227**: 680–685

La Porta CAM and Comolli R (1995) Over-expression of protein kinase C δ is associated with a delay in preneoplastic lesion development in diethylnitrosamine-induced rat hepatocarcinogenesis. *Carcinogenesis* **16**: 1233–1238

Lee SA, Karaszkiewicz JW and Anderson WB (1992) Elevated level of nuclear protein kinase C in multidrug-resistance MCF-7 human breast carcinoma cells. *Cancer Res* **52**: 3750–3759

Martiny-Baron G, Kazanietz MG, Mishak H, Blumberg PM, Kochs G, Marme' D and Schachtele C (1993) Selective inhibition of protein kinase C isoenzymes by the indolocarbazole Gö6976. *J Biol Chem* **268**: 9194–9197

Muller I, Janner A, Bruchel T, Niethammer D and Halliwell B (1997) Effect of concentration on the cytotoxic mechanism of doxorubicin-apoptosis and oxidative DNA damage. *Biochem Biophys Res Commun* **230**: 254–257

O'Brian C and Ward N (1989) Biology of the protein kinase family. *Cancer Met Rev* **8**: 199–214

Orr GA, Han EKH, Browne PC, Nieves E, O'Connor BM, Yang CP and Horowitz SB (1993) Identification of the major phosphorylation domain of murine mdr 1b P-glycoprotein. *J Biol Chem* **268**: 25054–25062

Palayoor ST, Stein JM and Hait WN (1987) Inhibition of protein kinase C by antineoplastic agents: implication for drug resistance. *Biochem Biophys Res Commun* **148**: 718–725

Posada J, Vicki P and Tritton TR (1989*a*) Protein kinase C in adriamycin action and resistance in mouse sarcoma 180 cells. *Cancer Res* **49**: 6634–6639

Posada JA, McKeegan EM, Worthington KF, Morin MJ, Jaken S and Tritton TR (1989*b*) Human multidrug resistant KB cells overexpress protein kinase C: involvement in drug resistance. *Cancer Commun* **1**: 285–292

Towbin H, Staethlin T and Gordon J (1979) Electrophoretic transfer of proteins from polyacrylamide gels to nitrocellulose sheets. *Proc Natl Acad Sci USA* **76**: 4350–4354

Yu G, Ahmad S, Aquino A, Fairchild CR, Trepel JB, Ohno S, Suzuki K, Tsuruo T, Cowan KH and Glazer RI (1991) Transfection with protein kinase C α confers increased multidrug resistance to MCF-7 cells expressing P-glycoprotein. *Cancer Commun* **3**: 181–189

Ward NE and O'Brian CA (1991) Distinct patterns of phorbol ester-induced downregulation of protein kinase C activity in adriamycin-selected multidrug resistant and parental murine fibrosarcoma cells. *Cancer Lett* **58**: 189–193

Wetsel WC, Khan WA, Merchentaler I, Rivera H, Halpen AE, Phung HM, Nagro-Vilar A and Hannun YA (1992) Tissue and cellular distribution of the extended family of protein kinase C isoenzymes. *J Cell Biol* **117**: 121–133

© Cancer Research Campaign 1998